# Existing evidence on the impact of climate risk on real estate valuations: a systematic map

**DOI:** 10.1186/s13750-026-00389-6

**Published:** 2026-06-23

**Authors:** Tommaso Piseddu, Fedra Vanhuyse

**Affiliations:** 1 Stockholm Environment Institute, Stockholm, Sweden; 2 Department of Real Estate and Construction Management, KTH – Royal Institute of Technology, Stockholm, Sweden; 3 Faculty of Economics and Business Studies, University of Ghent, Ghent, Belgium; 4 Faculty of Political and Social Sciences, University of Ghent, Ghent, Belgium; 5 Centre for Health and Sustainability, Uppsala University, Uppsala, Sweden

**Keywords:** Natural disasters, Economic losses, Climate risk, Transition Risk, Physical risk

## Abstract

**Background:**

Global climate change is set to create a future where natural hazards become more catastrophic and more frequent, with impacts on real estate expected to be substantial. At the same time, the transition to a low-carbon economy is reshaping asset values, with high-emission assets becoming less attractive.

**Methods:**

We adopted a systematic map approach to assess how the impact of climate risks, i.e., physical climate risks and transition climate risks, has been assessed in the academic and grey literature related to real estate valuation, published between 2014 and 2023. The retained corpus of 130 documents was coded according to a pre-defined coding framework, covering geographic distribution, evaluation methods, the type of real estate assets and impact assessment methods.

**Review findings:**

Across the 130 included documents (peer-reviewed and grey literature), the evidence base is dominated by studies that quantify physical climate risk effects on real-estate values, most often using econometric capitalization approaches (e.g., hedonic regressions, DiD, repeat-sales). The most frequently studied hazards are hydrological ones, especially coastal and fluvial floods, typically operationalized using official hazard maps (particularly in US case studies) or through proxies such as elevation and distance to from the shoreline.

A smaller subset of studies evaluates adaptation-relevant outcomes (e.g., damages via depth–damage functions) or uses alternative valuation variables such as asking prices, appraisals, or operating and maintenance costs in retrofit-focused works. Evidence on transition climate risks is present but much smaller in volume and is concentrated on a limited set of drivers, notably minimum energy performance requirements, energy labels and local externalities linked to renewable-energy deployment. This patterns partly reflects the study’s focus on transition risks explicitly linked to decarbonization.

In terms of geography, the literature is heavily concentrated in the United States, while European studies cluster in a few large economies (notably Germany, Italy, and the United Kingdom). The corpus is overwhelmingly focused on one- and two-family housing, with comparatively fewer studies examining multi-family housing and commercial real estate. A small number of papers touch on ecosystem or environmental amenities changes affecting values, including cases linked to invasive or alien species (e.g., algae blooms), but these studies remain relatively rare in the overall corpus.

**Conclusion:**

The systematic map highlights several priority gaps for future research and evidence synthesis. First, transition risks remain underexplored beyond a narrow focus on energy-performance regulation, energy labels and renewable-energy proximity; more work is needed on additional channels such as disclosure regimes, financing and insurance repricing, changing tenant demand, and stranded-asset dynamics. Second, compound and cascading risks are rarely analyzed explicitly, even though real-estate exposure often reflects interacting hazards and changing baselines; advancing datasets and methods that represent multi-hazard interactions is therefore a key priority. Third, the geographic evidence base is skewed toward the United States, and more comparable, data-rich studies are needed across Europe to strengthen external validity. Fourth, research remains focused on single-family housing, limiting transferability to urban contexts where multi-family and commercial assets, and different ownership, tenancy, and financing structures, are central. Finally, ecosystem-related valuation impacts appear in only a small portion of the academic corpus and are limited in grey literature, suggesting a need for clearer conceptualization and more systematic integration of biodiversity and ecosystem mechanisms in real-estate climate-risk assessment.

**Supplementary Information:**

The online version contains supplementary material available at 10.1186/s13750-026-00389-6.

## Background

Climate change is altering hazard regimes, with substantial evidence that many regions will face more frequent or more intense events such as heavy precipitation and floods, droughts, heatwaves, storm surges and tropical cyclones (1–11). These changes can affect food security (12), health, including psychological health (13, 14), and economic and financial stability (15, 16).

In finance, climate‑related risks are commonly grouped into physical risks and transition risks (17–21). Physical risks refer to direct impacts from acute events (e.g., floods, heatwaves, storms) and from chronic changes (e.g., sea‑level rise). Transition risks refer to the economic and financial consequences of the transition to a low‑carbon economy, including policy and legal changes, technology shifts, market re‑pricing and reputational channels (17, 20).

**Fig. 1:**
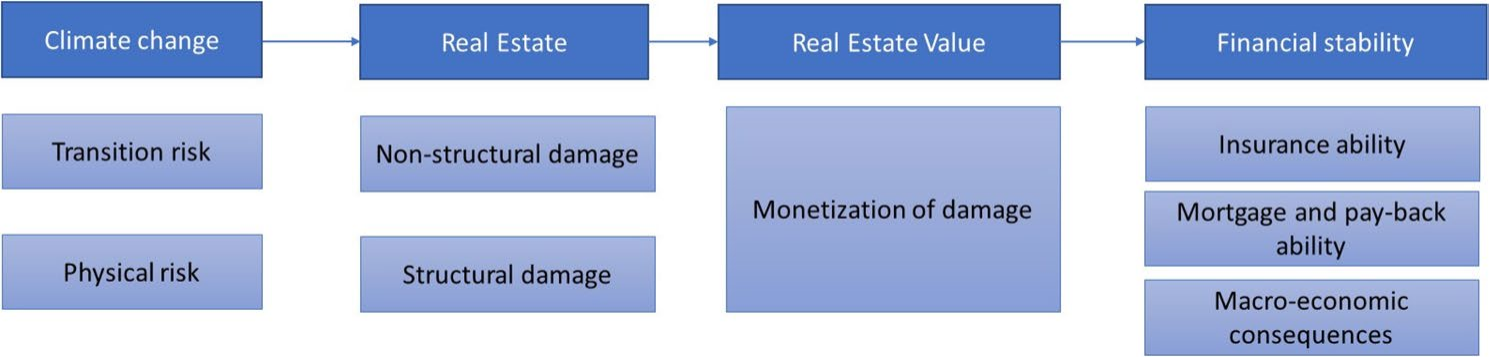
Linking climate change and climate risks to financial stability

Real estate is a particularly relevant sector for studying these risks: assets are location‑fixed, long‑lived, and often highly leveraged, which can amplify both asset‑level losses and wider financial‑system spillovers (Fig. 1). Real estate markets also represent a substantial share of global economic and financial wealth. In the EU, commercial real estate is valued at around USD 7.7 trillion, or about 43% of the Union’s GDP, with the North American market valued at USD 12.4 trillion (22). The global figure for global residential markets is estimated to be worth around US$286 trillion (23). In addition, real estate investments typically have longer time horizons and lower liquidity compared to other financial assets.

Most financial assets exposed to climate risk have shorter investment horizons than real estate assets. Treasury Bills, for example, have a maximum duration of 52 weeks (24) while stocks and funds are held on average between 1.1. and 4.8 years (24). Mortgages, by contrast, have a 30 years maturity (25).

Real estate assets are also often financed through debt. In Sweden, for example, mortgages represent about 83% of the total lending to households (26). As a result, shocks to real estate markets can propagate to the broader economy, particularly through effects on household consumption.

The long time horizon of real estate investments further increases their exposure to climate risk. Some hazards, such as sea level rise, unfold gradually and may not affect assets with short holding periods. However, longer time horizons increases the likelihood of experiencing low-probability events. For example. a 100-year flood corresponds to a 1% annual probability of occurrence. Over a 30-year period, however, the probability of experiencing at least one such event rises to approximately 22% (27). Taken together, the size of the real estate markets, their illiquidity, their connection to household finance and their long term investment horizons make the sector particularly relevant for assessing the economic consequences of climate risks.

The evidence base is both rapidly expanding and fragmented across hazards, geographies, asset classes and empirical methods. Previous research has described house prices as potential ‘barometers’ of risk (25, 28). However, efficient capitalization into prices depends on information being available and salient to market participants. Incomplete disclosure and information frictions can lead to mispricing and delayed adjustment (29, 30). This fragmentation is reflected in mixed empirical findings: while some studies show that climate risks are negatively capitalized in housing prices (31, 32), others find limited or no such effects (30, 33, 34). A systematic map can therefore help identify clusters of evidence and gaps, support future systematic reviews, and inform data and policy priorities for climate-risk assessment in real estate markets.

In this paper, we report a systematic map of literature on climate risks and real estate valuation. We first present the objectives and summarize the methods and any deviations from the registered protocol (35). We then describe categorized evidence on where studies are located, which risks and assets they cover, and which identification and valuation approaches they use. We conclude with implications for research, data needs and policy practice.

## Objective of the review

The objective of this systematic map is to characterize and compare (1) the peer‑reviewed evidence and (2) relevant grey literature on how climate risks are operationalized and linked to real estate valuation outcomes. Specifically, we map evidence on:

- how studies define and measure climate risks (risk type and, within physical risks, hazard types; within transition risks, policy/legal, technology, market, reputational channels);
- which real estate asset classes and valuation outcomes are analyzed (e.g., transaction prices, rents, expected damages, cash‑flow variables);
- where and when studies are conducted (geographic coverage and study period);
- which data sources and analytical approaches are used to identify exposure and estimate impacts (e.g., hazard maps, energy labels/certifications, econometric designs, damage models).

These mappings support two primary questions:

- Q1: How have climate risks for real estate markets been described and operationalized in the literature?
- Q2: How have climate risks been reported or estimated to affect real estate valuation outcomes?

Below, we develop the components of the research questions in the PECO (Population, Exposure, Comparison, Outcome) structure.

## Methods

The methods adopted to retrieve the relevant literature have been presented at length in the protocol (35) and will be briefly summarized here.

### Search for articles

We followed the ROSES reporting guidance (36) and completed the ROSES template (see protocol (35)). Searches were conducted in summer 2023.

We searched 3 sources: Scopus and Web of Science for peer‑reviewed journal articles, and Overton for grey literature (37, 38). Scopus and Web of Science are the largest academic databases (37) and, compared to, for instance, an alternative like Google Scholar, make it straightforward to extract information in bulk and do not limit the number of results displayed. Overton is the largest repository of grey literature, including reports, working papers, and regulatory documents (38).

We restricted results to English‑language documents published between 2014 and 2023 (inclusive). Limiting the search to the most recent decade is a common practice in systematic mapping (39, 40), and reflects both data availability and methodological advances. More recent studies benefit from larger transaction datasets, improved methods, and stronger policy relevance. For the academic databases we restricted results to published peer-reviewed journal articles. For Overton, results were ranked by the platform’s relevance score, and we downloaded the top 1,000 records.

The search string uses a terminology established in the economic and financial climate-risk literature (20, 41–44). We deliberately focused on the term climate risks to target studies explicitly linking climate change to economic outcomes in real estate. Regulatory bodies and international financial institutions, for example, refer to the negative economic and financial consequences of climate change as “climate risks”, including the European Central Bank (45), the Bank of England (46) or the European Environment Agency (47). The string was designed to be inclusive while remaining aligned with the scope of the study (48), allowing other specifications such as “climate-induced risks” or “climate change risks” to be considered. Search performance was benchmarked against a list of 50 relevant studies (35), following an established procedure in systematic maps and systematic literature review studies (49, 50). Real estate is an often used in systematic literature review and deemed sufficient to collect the relevant literature (51–53). The search string retrieved 75% of these studies, consistent with comparable systematic mapping exercises (54–56). The final search strings are reported below for transparency:

Scopus: ALL (climat* AND risk* AND (value OR economic OR financ*) AND (real AND estate OR building*)) AND (LIMIT-TO (DOCTYPE, “ar”)) AND (LIMIT-TO (PUBYEAR, 2014) OR LIMIT-TO (PUBYEAR, 2015) OR LIMIT-TO (PUBYEAR, 2016) OR LIMIT-TO (PUBYEAR, 2017) OR LIMIT-TO (PUBYEAR, 2018) OR LIMIT-TO (PUBYEAR, 2019) OR LIMIT-TO (PUBYEAR, 2020) OR LIMIT-TO (PUBYEAR, 2021) OR LIMIT-TO (PUBYEAR, 2022) OR LIMIT-TO (PUBYEAR, 2023)).

In Scopus we used the term ALL() to maximize sensitivity (capturing titles, abstracts, keywords and other indexed fields); this choice increases retrieval but does not reduce coverage relative to TITLE‑ABS‑KEY(), and relevance was ensured through multi‑stage screening (see below).

Web of Science: TS=(climat* AND risk* AND (value OR economic OR financ* ) AND ((real AND estate) OR building* )) AND PY=(2014–2023).

After the search, a filtering was applied to only retrieve results in English and within the limits of the geographies mentioned below among the eligibility criteria.

### Screening process

Results were downloaded as comma-separated values files (.csv) for bulk processing. They were then imported in Python as *pandas.DataFrame* using the library *Pandas* (57) and merged with the *pandas.DataFrame.merge()*, function specifically designed to perform a database-style join. The titles of the results were then rendered to lowercases using the .*lower()* built-in Python method. This step ensures that the same titles spelt differently with lower- and uppercase letters were identified as duplicates. *pandas.DataFrame.duplicated()* was then used to identify duplicated titles. The academic literature from the two sources was merged into a single corpus, from which duplicates (*n* = 220) were removed.

The merged corpus was then split evenly among the reviewers. The academic literature was screened at the title, abstract and full-text level. Screening at the title level first and at the abstract level after is common practice in systematic literature reviews and systematic maps (58, 59). At the title level, records were excluded only when they were clearly out of scope. Where relevance was uncertain, records were retained for abstract screening. Abstract screening then applied the full eligibility criteria. Full‑text screening and coding were subsequently performed for all retained records. The screening process for the grey literature differed because these documents generally did not include abstracts, and many lacked a summary. Grey literature was therefore screened at the title and at the full-text levels only.

To assess reviewers’ consistency, both authors independently screened a random sample of 200 titles from each academic database. A Cohen’s kappa (60, 61) was computed: 0.64 for the Web of Science literature and 0.66 for Scopus, exceeding a commonly employed threshold of 0.60 for ‘substantial’ agreement in evidence synthesis workflows (55, 61–63). For abstract screening, both authors independently screened a 10%, randomly allocated, sample and achieved a Cohen’s kappa score of 1, indicating perfect agreement. At full text, we double‑coded a small pilot set and resolved any discrepancies through discussion.

**Fig. 2:**
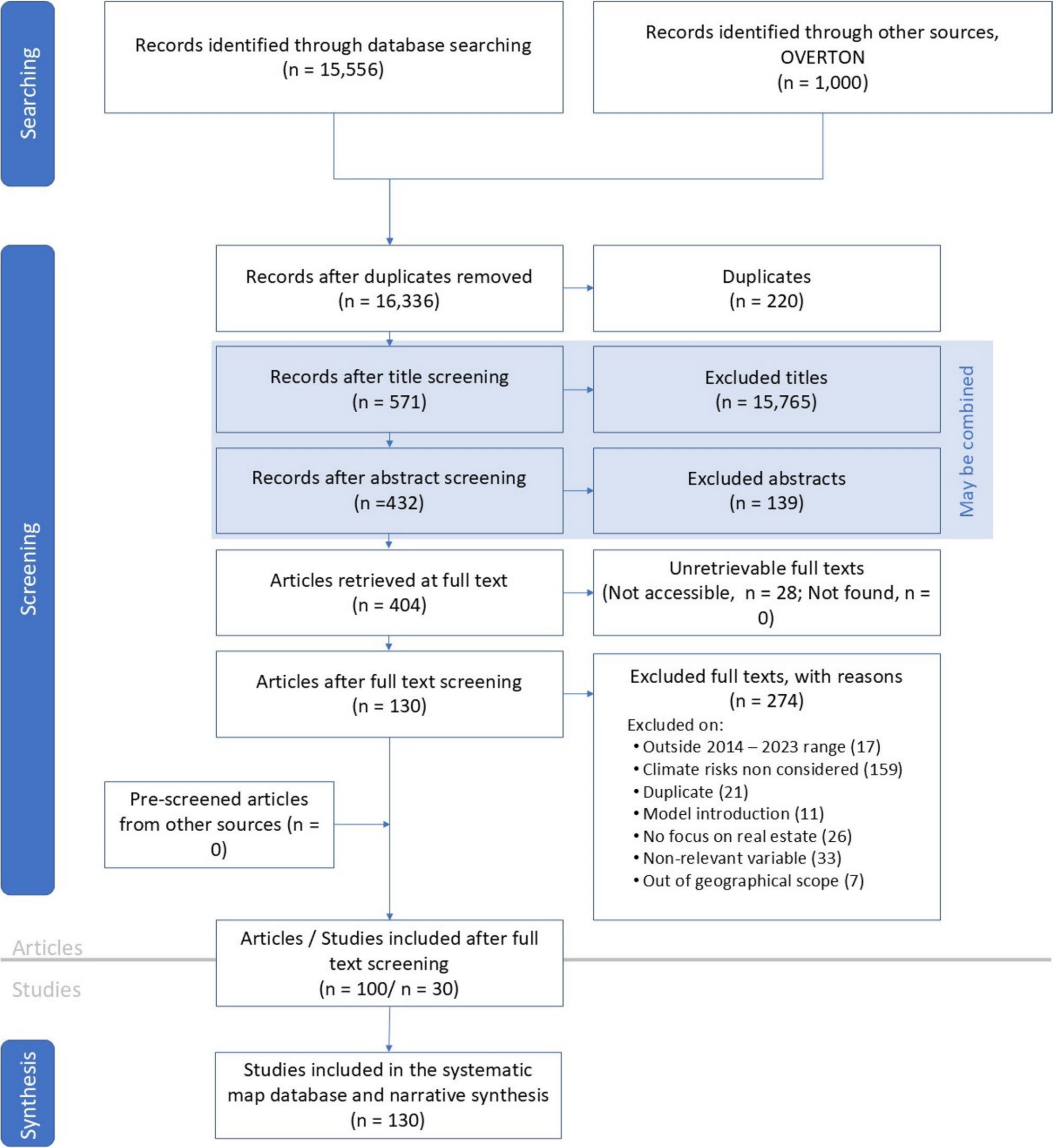
Summary of the search and screening process

The retained final corpuses consisted of 100 academic articles while 30 documents from the grey literature. The full screening process is summarized in Fig. 2. For full-text screening, the figure provides specific reason for exclusion, beyond the elements of the PICO/PECO framework. Most exclusions at full text are due to duplicate results from Overton. This is often because documents are stored in the database with slightly different titles, making it difficult to identify duplicates without reading the full-text. Most publications excluded for being outside the adopted time range also came from Overton, with (64) being the only study from the peer-reviewed literature excluded at the full-text level for this reason. One relevant example from Overton is (65), which is stored on the database as being published in 2014. Only the full-text analysis reveal that the actual publication date is 2008. Studies (66) and (67) are examples of studies excluded only at the full-text level because they fell outside of the geographical scope of this review. From the title and abstract screening, it was not possible to determine that these are case studies in Australia and Nigeria. The majority of the exclusions pertain studies that do not address the research topic of climate risks, such as (68, 69). Studies with no focus on real estate assets were also excluded from the final corpus (70) as were studies designed exclusively to introduce models (71).

### Eligibility criteria

The eligibility criteria for the documents are presented in the following PECO (Population, Exposure, Comparison, Outcome) framework:

- Population: real estate assets (Offices, Industrial and Logistics, Retail, Residential, Healthcare, Hotels, etc.). Geographically, we filter to Europe and North America (USA and Canada).
- Exposure: exposure to climate risks, whether physical climate risks or transitional climate risks, actual, based on a model, on future projections or on valid assumptions.
- Comparison: pre- and post-comparisons with the value of the asset, comparable assets with similar characteristics in the proximity that are not exposed to the risks, value of the asset in the absence of exposure to risks.
- Outcome: a variation in the value of the assets that are subject to any form of climate risk; an estimate in absolute monetary units, monetary units per m^2^, or a percentage variation.

We focused on results from North America, including the United States of America (USA) and Canada, and Europe, consistent with our registered protocol (35). These two parts of the world are also the ones where the lowest level of insurance gaps are observed (72). Access to detailed, precise and reliable damage estimates is a prerequisite for an accurate estimation of the impact of climate risks on the real estate sector. Housing prices can also be more readily analysed in these geographical areas because market information is relatively transparent (73). The availability of large datasets, especially in North America, where Zillow has over 400 million observations (74), guarantees the possibility to collate evidence from robust results. Finally, European and North American countries also often rank at the top of vulnerability and readiness indexes, suggesting a relatively long history of dealing with climate risks that research can build upon (75). By contrast, the scarcity of real estate transaction datasets for other large real estate markets, such as the People’s Republic of China, limits the evidence base relevant to this systematic map (76, 77). The composition of the Advisory Board behind this study, which served as a venue for exchange of ideas and results, also influenced the decision to restrict the geography to markets where the members have more first-hand experience. Similarities in property rights also shaped the decision to restrict the geographical scope to North America and Europe. The Joint Research Centre (JRC) shares the International Property Rights Index on a yearly basis, making it possible to compare the political and legal environments that shape physical property rights, European and North American countries, with the minor exceptions of a few English-speaking countries and Japan, consistently rank at the top of the index, revealing similar standards in protecting property rights and therefore enabling a full functioning housing market (78). Others who have attempted to produce property right indexes using different methods have reached similar conclusions, reinforcing the similarity between North American and European countries (79). Lastly, cultural proximity, another aspect that is considered relevant for understanding how markets function, provides an additional justification for focusing on North America and Europe. The seminal work by (80) clusters countries according to values and culture. The average discriminant probability of classification reported in the study suggests, for instance, that the second most adequate cultural classification for Anglo cultures is Latin European cultures. Likewise, Latin European countries are found to be the closest to Anglo Cultures. Germanic and Nordic European cultures provide an additional example of clusters that are closed to each other. Overall, the pattern reveals significant degrees of proximity between the cultures found in Europe and those found in North America.

For the operationalisation of transition risks, consistent with established climate‑risk frameworks (17, 81), we coded transition‑risks studies when valuation outcomes were linked to drivers of the low‑carbon transition, including policy/legal changes such as energy‑performance standards, technology shifts and market re‑pricing, including renewable energy deployment, energy labels/certifications when discussed in a mitigation or transition context (45). Purely financial or engineering analyses of energy retrofits that did not reference climate mitigation, decarbonisation or transition drivers (e.g. (82)), were considered outside the scope of this climate‑risks map. We note that this operational definition may under‑capture economic studies where transition is implicitly priced without explicit transition framing.

### Data coding strategy

From the retained corpus (*n* = 130), we extracted and coded the variables listed in Table 1, following the pre-defined coding framework in the protocol (35). Coded variables were summarized descriptively (counts and cross‑tabulations) and visualized in figures. We report patterns separately for peer‑reviewed and grey‑literature corpora because the sources differ in document types, retrieval strategy (Overton top‑ranked results) and reporting conventions.

**Table 1:** Variables and information extracted from the final synthesis corpus

Variable	Operational definition / examples (coding option)
Bibliometric information	Source (Scopus, Web of Science) Type of document (Journal Article) Year Authors Title DOI Source title Abstract
Type of climate risk	Physical risk Transition risk
Type of physical risk	Inspired by (69) and (70): • Temperature-related chronic: changing temperature, heat stress, temperature variability, permafrost thawing • Temperature-related acute: heat wave, cold wave/frost, wildfire • Wind-related chronic: changing wind patterns • Wind-related acute: cyclone, hurricane, typhoon, storm (blizzards, dust and sandstorms), tornado • Water-related chronic: changing precipitation patterns and types; precipitation and/or hydrological variability, ocean acidification, saline intrusion, sea level rise, water stress • Water-related acute: drought, heavy precipitation (rain, hail, snow/ice), flood (coastal, fluvial, pluvial, ground water), glacial lake outburst • Solid mass-related chronic: coastal or soil erosion, soil degradation, solifluction • Solid mass-related acute: avalanche, landslide, subsidence
Transition risk channel	• Policy and legal risks • Liability risk • Technology risk • Market risk, and • Reputation risk
Geography	(name city, region and/country)
Exposure identification	Inspired by (38), including: • Integrated Assessment Model (IAM) • UKCCRA • WorldRiskIndex
Impact assessment method	Econometric design (e.g., Hedonic OLS, repeat sales, DiD, matching), simulation/ discounted cash-flow; depth-damage models; Monte-Carlo simulation; other (71, 72)
Impact/ valuation outcome	Reparation costs Adaptation costs Lowered rent opportunities
Asset class	Residential (single-family; multi-family) Commercial (offices; industrial; retail; hotels), and Others
Timeframe of the analysis	Forecasted, future damage or actual, historical damage (year of the forecasted value or actual damage)
Recommendations	Suggestions put forward in the paper related to, for example, the calculation methodology, regulation around climate risk and real estate valuation, mitigation of climate risk, etc.
Scope for further analysis	Research gaps mentioned in the paper
Further relevant notes	Any other relevant statement in the paper that could be useful but does not fall under the different categories of the coding framework.

Source: authors’ own elaboration

### Stakeholders engagement

The goal of this map was to identify trends and gaps both in the grey and in the peer-reviewed literature investigating the impact of climate risks on real estate valuations. All the elements of our strategy, from the search string to the final presentation of the results, benefited from the continued engagement with experts in the field through the Advisory Board of the Vinnova-funded MAVERIC project (Material Asset Valuation: Estimating Risks and Impact of Climate Change). Many of these participants represented banks, insurance companies, real estate owners and consultants within the real estate sector. The Swedish Hydrological and Meteorological Institute (SMHI) was also part of this exchange. In addition, the methods and the preliminary results of the mapping were presented at the Malmö Real Estate Research Conference 2024 and benefited from participants’ comments and inputs.

### Deviations from the protocol

We report the following deviations from the registered protocol (35). First, the reviewers team decreased from three to two reviewers. Second, due to resource constraints, inter‑reviewer agreement at abstract screening was assessed on a 10% random sample rather than on 200 abstracts as initially planned. Third, as the academic literature databases employed in this study do not allow to set precise dates in a day/month/year format, the number of results retrieved using the same search string was higher than what stated in the protocol. We argue that while this indeed constitutes a deviation from the protocol, the coverage of the study actually improved. Fourth, the search string employed for the academic literature search allowed articles from 2013 to enter the final corpus of the results. We did not re‑run searches; instead, all 2013 records were removed during screening to maintain the intended 2014–2023 coverage. Only one study from 2013 was included by mistake in the final corpus for evidence and was excluded at the full text level. This is outlined in Fig. 2 and counted among the studies that were “Excluded full text with reasons”, in the last step of the screening phase Finally, we noticed during coding that a small number of studies addressed environmental amenities/ecosystem change without framing these as physical or transition climate risk. These were recorded as ‘other’ but are not treated as a core evidence gap because our search strategy was not designed to systematically capture biodiversity- or nature‑related risk.

## Review findings

We report the findings from the mapping along the variables presented in Table 1, covering first the temporal and geographical trends in the number of results retrieved. We then progress by discussing the types and numbers of climate risks investigated and conclude by showcasing the methods, both to estimate the exposure and to estimate the impact, and the types of real estate assets.

### Temporal trends

**Fig. 3:**
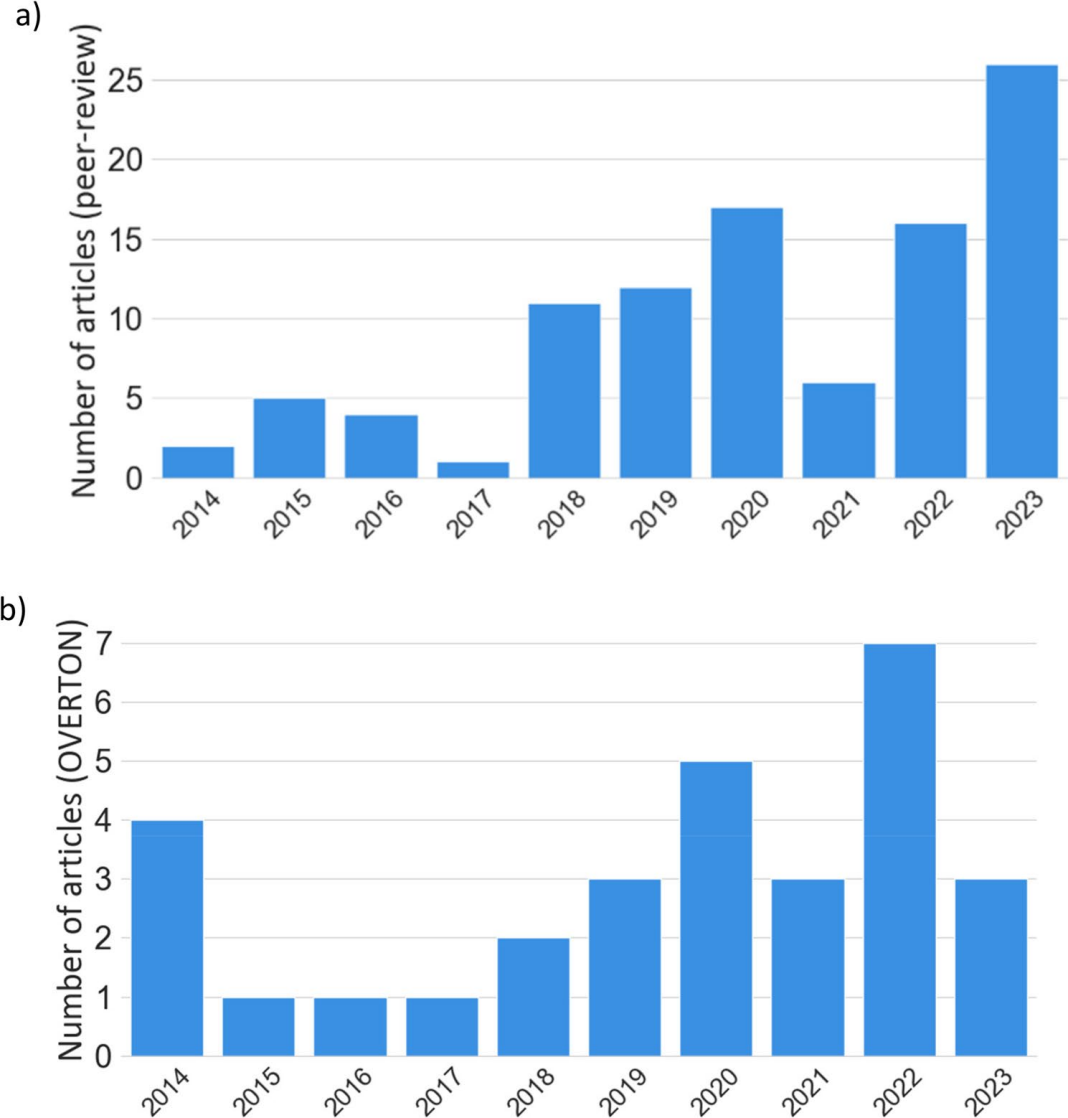
Number of peer-review publications (a) and OVERTON (b) per year in the final corpus

Figure 3 shows publication trends over time for the peer‑reviewed (Fig. 3a) and grey‑literature (Fig. 3b) corpus. Peer‑reviewed publications increase over the period (83), with a noticeable dip in 2021. This may reflect topic‑specific dynamics or broader COVID‑19‑related delays in data collection, fieldwork and peer‑review cycles. A similar trend is less evident in grey literature, with 2014 displaying more results than any of the following years up to 2019. Over the years, the case‑study focus of many articles suggests that high‑impact events can stimulate research attention, although our map does not attempt to estimate causal links between event occurrence and publication counts. Indeed, it is possible that certain events, especially those receiving large media attention, may have motivated researchers to investigate the topic. The significant increase in number of grey-literature outputs in 2022 could also be linked to the COP26 meeting in Glasgow in 2021. At the time of its occurrence, it was the largest COP ever by number of attendees, produced significant policy implications beyond what had been initially expected and is still regarded as one of the most meaningful COP events in history (84).

The reader should be cautioned that, as the search was conducted in summer 2023, the number of results from this year does not represent a full account of the relevant research published during that year. It is also relevant to note that, for both types of literature, 2017 represents the year with the lowest number of relevant documents. For the peer-reviewed literature, which is mostly made up of case studies, this could reflect the trend observed in the economic damage produced by natural disasters. All the years between 2013 and 2016 recorded significantly lower-than-average economic losses from natural hazards, with 2014 and 2015 representing, respectively, the lowest and second lowest figures from 2010 to today (72). The absence of significant natural disasters, compared to a long-term perspective, could potentially help to explain the lower interest in the topic. The lower-than-average trend in grey literature could well be justified by a quieter interest for environmental topics, as, for instance, exemplified by the low attendance at international meetings such as COPs in this period (85).

**Fig. 4:**
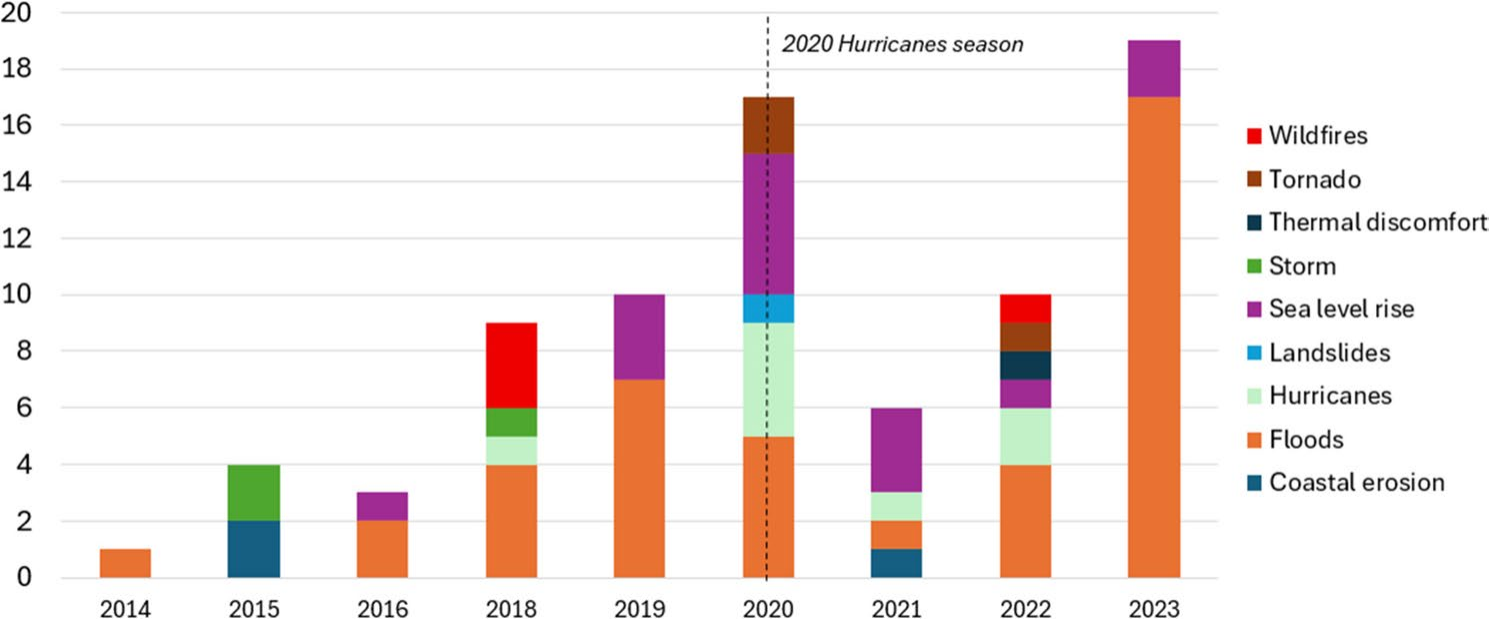
Breakdown of the peer-reviewed publications by natural hazards and by year

Figure 4 summarises how physical‑risk studies are distributed across hazard types by publication year. At this stage, we do not distinguish between different types of floods and instead treat flooding as a single hazard category; returning to this distinction later in the manuscript. Hurricanes began to appear more prominently from 2020, probably due to the frequency with which this natural hazard occurred in the USA (86), the only country from which we retrieved results on hurricanes. At least one study (87), , uses the 2017 hurricane Sandy as a case event to assess the impact on housing prices. While the 2010 fire season has been judged “below normal for number of reported wildfires at 94% of the 10-year average […]” [88], it is interesting to notice that 2 (50%) of the retrieved studies focusing on wildfires use events from 2010 to base their case studies (89, 90).

### Geographical distribution

**Fig. 5:**
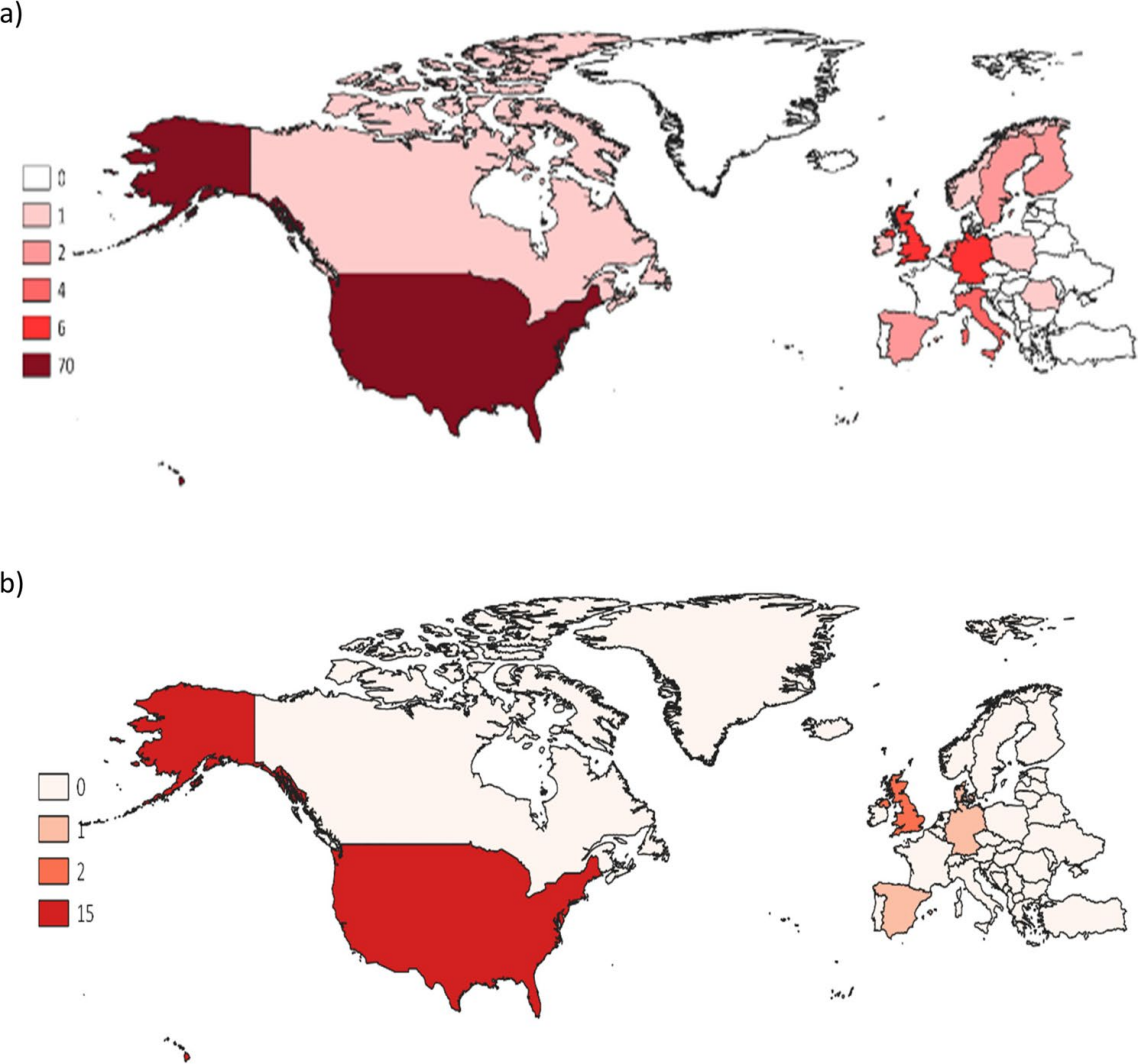
Geographical distribution of the peer-reviewed results (a) and of the grey literature (b)

Most of the peer-reviewed articles focus on case studies in the USA (*n* = 70), with a particular focus on Florida and the Miami-Dade County, the hotspots of research on hydrometeorological hazards. The USA has consistently experienced the largest economic damages from natural hazards (72), and its commercial real estate value is the highest in the world (22). Of the remaining 30 academic studies, six were conducted in Germany and in the United Kingdom, four in Italy, two in Spain, Sweden, the Netherlands and Finland, while Canada, Ireland, Norway, Poland and Romania all contribute to the final corpus of studies with a single document each. For the grey literature, while the European Central Bank is the single most prolific author, most studies focus on the USA (*n* = 15) in this group too. Figure 5 displays the geographical focus of the corpus, broken down for the academic peer-reviewed literature (Fig. 5a) and for the grey one (Fig. 5b).

### Types of climate risks assessed

**Fig. 6:**
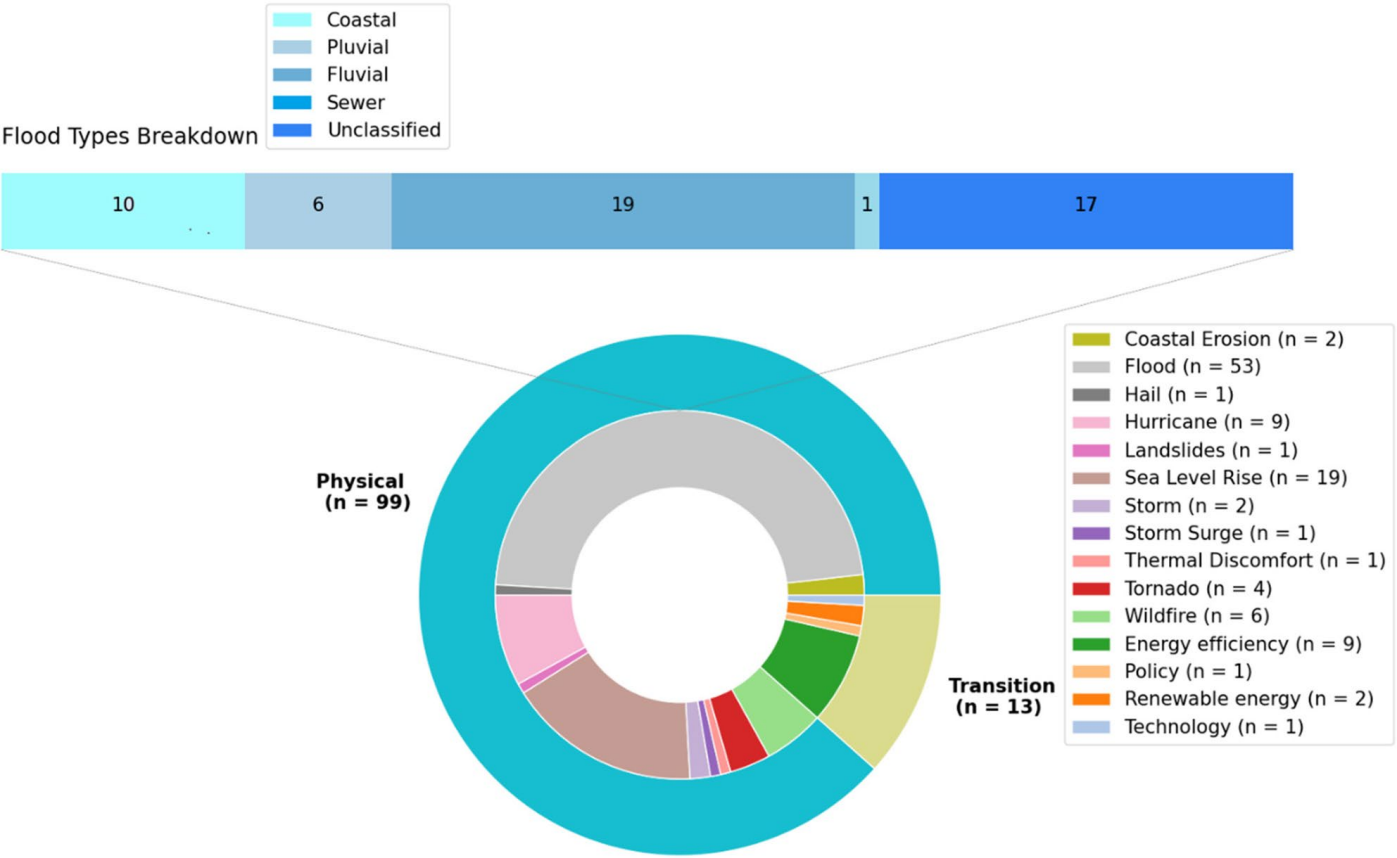
Mentions of climate risk in peer-reviewed publications by type of driver. The same document may mention more than one risk; therefore, the total count of risks exceeds the number of documents. The distinction between physical and transition follows the economic literature. The physical risk category includes studies on natural hazards, while the transition risk category captures measures adopted to mitigate and adapt to climate change

Within the academic corpus, 83 articles investigated physical climate risks (natural hazards) and 13 transition risks linked to the low-carbon transition. A small number of additional studies (*n* = 4) address environmental amenities or ecosystem changes affecting local property values without framing these as climate‑risk drivers; we record these as ‘other’ and do not interpret their low count as evidence of a systematic gap because our search strategy was not designed to capture biodiversity/nature‑risk terminology (Fig. 6).

Floods were by far the most covered hazards (*n* = 53), with fluvial flooding (*n* = 19) and coastal flooding (*n* = 10) most frequently researched. About 17 of the documents did not disclose how floods occurred, i.e., whether these originated from a river (riverine flood), from a heavy rainfall event (pluvial) or from the coastline (coastal floods). Sea level rise is the second most frequently investigated hazard (*n* = 19), followed by hurricanes (*n* = 9). The dominance of hydrological hazards in our corpus is not surprising, as disaster risk management practices have focused mostly on this type of event (91). Hydrometeorological hazards have also been the costliest type of natural hazard in the last 20 years (72), and flood risk maps are readily available, making it more feasible to address several aspects related to such a hazard.

While flooding is the only event that spans all geographies (92–95), some hazards are exclusively associated with specific regions or countries. For example, sea level rise is addressed only in studies conducted in the USA and Norway (96–99), confirming these two countries as among the most at-risk (100, 101). Despite the increase in damages due to the growing occurrence of convective storms (often referred to, or linked to, cloudburst events (102, 103)), with 2023 marking a new record for damages in North America (72, 104), we found only three studies focusing exclusively on pluvial flood events in urban areas (105–107).

Hurricanes and tornadoes are two US-only hazards in our dataset, and their geographic distribution reveals the inherently different characteristics of these two events. Tornadoes are smaller in size and tend to generate inland, confirming the results of our dataset with three studies coming from Oklahoma (108–110) and the fourth one relying on data from the South, the West, the Midwest and the Northeast of the US (111). The nine case studies focusing on hurricane events all rely on evidence coming from the US East Coast, with five studies from Florida (112–116), two from New York (87, 116) and one from North Carolina (117). The lack of studies on the impact of tornadoes in Europe reflects the climatic conditions of a continent that is, on average, hit by between 1 and 3 strong tornadoes every decade (118).

Wildfire studies in our corpus come exclusively from the USA. In particular, the Mountains region in the country seems to be the most affected: 3 studies are based on wildfires that occurred in Colorado (90, 119, 120), and then one each for Arizona, California and New Mexico (89, 120, 121). The presence of so many cases from the USA does not imply that wildfires do not represent a risk in Europe, but it could be explained by the fact that the USA has had some of the costliest and largest wildfires ever recorded and has the highest fire insurance uptake in the world (72), a circumstance that guarantees detailed and complete datasets for research purposes.

Among studies on transition climate risks (*n* = 13), we find topics on energy efficiency (*n* = 9) and renewable energy (*n* = 2) to be the most frequently investigated. The former group is comprised of studies that investigate the presence of a green premium, i.e. the additional value that the market attributes to assets with low energy demands (122–125), while the latter consists of studies in which the initiative to reduce housing energy emissions is tackled at the source, by replacing the source of energy production (126, 127). are the only two studies in this second group, and they both focus on how switching the local energy plant to more sustainable sources may impact housing values in the surrounding area. Technology and policy transition climate risks are addressed in the same study, where technology risk is intended as the adoption of different forms of heating and electricity supply sources and policy risk is introduced as a potential carbon tax (128). The limited number of studies dealing with the consequences of technology and policy adoption may be due to the uncertainties that characterize them: the studies are drafted as scenario analyses. Energy efficiency and renewable energy studies are instead based on actual data from transaction datasets, a feature that could contribute to the robustness of the analysis. Most policy documents obtained through the search in Overton provide general considerations around what they refer to as climate risks and delve into the topic by providing several examples of both physical and transition risks (129–131). These documents most often do not serve as case studies to advance research knowledge but rather provide an overview or a regulatory discussion that may be of interest to financial actors.

### Climate risk exposure assessment methods

Most studies estimating the impacts of hydrological hazards identify exposed assets using official hazard maps produced by public authorities (*n* = 40). This approach is particularly prevalent in US-based case studies (132, 133) where the Federal Emergency Management Agency (FEMA) has produced flood hazard maps for decades (134) and the National Oceanic and Atmospheric Administration (NOAA) has made sea level rise projections publicly available (135, 136). These products have been periodically updated as hydrological modelling has improved and as changing climatic conditions have altered risk profiles, creating opportunities for researchers to examine how revisions in mapped exposure translate into changes in housing prices, e.g., when properties shift from “at risk” to “not at risk,” or vice versa (33).

In addition to official hazard maps, several studies rely on proxy-based or model-based approaches to define exposure. Where sea-level-rise exposure is the focus, risk is often proxied using elevation and distance to the shoreline (99, 137–140). For pluvial floods, researchers frequently rely on hydrological models to delineate exposure when official maps are unavailable or considered outdated or insufficient (105–107). This is often justified by the observation that many existing maps do not capture pluvial flooding, despite increasing rainfall intensity, a concern also highlighted in the broader disaster risk management literature (141).

A further set of studies identifies exposure through observed past events (*n* = 11), using historical flood extents or event indicators to define affected areas (92, 111, 115, 142–144). One example uses the boundaries of North Carolina’s state buyout programme, an administrative indicator of flood-prone locations, to define exposure (93). This approach allows researchers to capture realised risk rather than modelled or anticipated exposure, and is particularly useful where historical event data are detailed and spatially explicit. However, it may also conflate exposure with realised damage, and may be sensitive to the specific characteristics of individual events, limiting comparability across contexts.

Finally, some papers use proximity-based measures to hazard “hotspots” or to adaptation interventions, particularly when studying measures such as fuel treatments for wildfire risk (112), beach nourishment to mitigate coastal erosion (145), seawall construction (146), or invasive species that degrade local environmental amenities (147). In these cases, distance functions are used as proxies for both risk and protection, capturing how perceived or actual exposure decays spatially. This approach is often adopted when direct measures of risk are unavailable, but it introduces assumptions about how risk is spatially distributed and perceived by market actors. It also allows for the analysis of adaptation measures as quasi-treatments, linking physical interventions to changes in property values.

### Elements of real estate values in focus

Final sales prices represent the most frequently employed measure to investigate the impact on assets’ values (*n* = 75), partly because sales prices can be easily accessed in large quantities. One study (126), for example, analysed well over one million sales transactions. The final sales price has been traditionally used in real estate valuation to test for the impact of new information (90, 148). For example, several studies investigated how economic actors process information by comparing the impact of flood risk maps and actual flood events (92, 144). A further paper (149) focuses on value appraisals, investigating whether the cost advantages of green buildings influence the negotiation between the buyer and the seller. Standard economic theory assumes that transaction prices reflect all available information, including exposure to climate risks (150), and are also considered to provide a good insight into the bargaining process between the buyer and the seller (151), with the latter potentially stressing the need to reduce prices in the presence of exposure to climate risks. It is not however uncommon for some of the studies in our corpus to make use of other variables such as asking prices (152).

At the same time, a subset of studies uses alternative dependent variables. Some studies (*n* = 14) focus on realised or expected damages, particularly in applications using depth–damage functions (97, 153, 154). Finally, operation and maintenance (O&M) costs appear only in studies evaluating energy-retrofitting measures (128, 155, 156).

### Impact assessment methods

For the estimation of the impact on sales prices, a linear hedonic price model (157) is by far the most common approach in our corpus (*n* = 39). Hedonic models are well suited to real-estate applications because they treat property prices as the sum of implicit prices for a bundle of heterogeneous attributes, allowing each characteristic to be assigned a marginal contribution to value (157). Empirically, this typically involves estimating an Ordinary Least Squares (OLS) regression where the dependent variable is the transaction price (or its logarithm), and the independent variables capture structural, locational, and risk-related characteristics.

The second most common approach is difference-in-differences (DiD) (*n* = 18). DiD is a quasi-experimental design in which the researcher does not determine the timing of the “treatment,” but instead exploits an external change or event to estimate its effect on an outcome. The method compares changes in the outcome for a treatment group (exposed) relative to a control group (unexposed), before and after the event, using the control group as a benchmark under the assumption of parallel pre-trends (27, 158). This structure makes DiD particularly relevant for climate-risk studies. For example, the event period can coincide with the publication of flood-risk maps: prices for properties newly identified as exposed can be compared with prices for similar but unexposed properties before and after map publication, when risk information becomes salient (29). Analogously, DiD can be used to study transition-related shocks, such as the announcement of a wind power plant construction: properties in the affected area constitute the treatment group, while comparable properties outside the area serve as controls (126).

A third common approach is repeat-sales analysis (*n* = 14), which compares the price of the same property across multiple transactions. In practice, repeat-sales models typically regress the change in log prices between two sales on covariates of interest (159). Because the dependent variable is a log difference, the estimated effects are interpretable as impacts on returns (percentage price change) rather than on levels. Importantly, a negative coefficient on an exposure variable therefore does not necessarily imply negative returns, but rather returns that are lower, on average, than those for unexposed properties. The principal advantage of repeat-sales is that it differs out time-invariant property characteristics (e.g., size), since each asset is effectively compared to itself. The main limitation is data intensity: the approach requires large datasets with many properties observed in at least two transactions. This is reflected in the longer time spans typically used: repeat-sales studies in our sample cover, on average, 21.75 years, with (160) using a 34-year period, whereas DiD studies have a shorter average time horizon (approximately 15 years).

Beyond econometric capitalization designs, depth–damage curves are also used extensively (*n* = 12). This engineering-based approach estimates a function linking flood depth to monetary damages (30, 97). It represents a standard in sea-level rise and fluvial flood applications (158, 159) and has increasingly been applied to pluvial flooding, although authors note that depth–damage functions are more developed for fluvial and coastal flooding than for pluvial-induced flooding (106).

Other methods used in the corpus include generalized linear models (161), discounted cash-flow modelling (156), and matching approaches (93, 121, 132, 162).

### Assets class

The methods used to estimate the impact of climate risk on assets’ values, we argue, are necessarily influenced by the type of assets and the valuation outcomes considered. Econometric models, the most common approach employed in our corpus, benefit greatly from large sample sets given their reliance on asymptotical assumptions (163, 164).

As large datasets exist for residential assets, the largest building stock and the most often traded compared to other types of assets, residential assets represent the largest category of real estate assets (*n* = 90) in our corpus, followed by commercial/office assets (*n* = 7). The datasets employed to address the research question on climate risks’ impacts on commercial real estate assets can be as small as 160 observations (149). In several instances, the impact is estimated through a theoretical model and only one single representative asset constitutes the basis for the analysis (128). Some studies focus generally on buildings without specifying the purpose they serve, while others combine multiple types in the same dataset (107, 154, 165). Multiple types of assets occur in studies dealing with flood risks, utilising the depth-damage curve approach. This approach incorporates asset typology (e.g., residential, commercial) as a key variable to calculate estimated damages (165–167).

Within residential real estate, most studies are limited to single-family houses, as explained by (109, p.730): “*Although exceptions exist*,* economic investigations of housing prices typically focus on standalone (i.e.*,* “single-family”) property housing markets”* and (125, p.212): “*we restricted our analysis to homes identified as a single family*,* omitting potential multi-family dwellings as is standard in much of the hedonic literature*”. The heavy focus of the literature on single-family houses is attributed by (132) to the fact that single-family buyers and condo buyers have inherently different preferences and process information in different ways, hinting that any exposure to climate risk, in particular transition ones, should be better reflected in the former category.

### Limitations of the map

The limitations of the map, mainly related to our search string, and the geographic and temporal dimension of our analysis, are addressed in this section. Firstly, while we tested our search string for comprehensiveness, as explained in our protocol (35), the terms selected for our search string could have resulted in omitted publications. The term “buildings”, for example, generated several irrelevant results, despite the Boolean operators that constrained the results to the presence of other terms too. However, such a term is still a standard in recent literature reviews focusing on real estate matters (168–171), hence we opted to include it. Secondly, the geographical and temporal limits affected the retained results. This leaves a gap in the global understanding of climate risk that we hope future research, with better access to data, will be able to fulfil. As argued in the systematic map protocol (35), the inclusion of the USA guaranteed that a good number of events are covered, given several billion-dollar economic loss events since the beginning of the century (72). Limiting the search to the last 10 years corresponds to an increased magnitude and the intensity of natural hazards in the last years: seven years of the 2014–2023 period have experienced more billion-dollar economic loss events than the average 2000–2023, with 2023 marking a record of 66 billion-dollar economic loss events (72). We acknowledge that excluding transition climate risks studies that did not make any direct connection with an attempt to reduce carbon emissions may have reduced the presence of such studies in our final corpus. Lastly, the type of underlying assets in the retrieved studies limit the possibility for the conclusions of this systematic map to inform policies on commercial real estate. While we identify this as a substantial gap in the previous literature, it also limits the external validity of our results.

### Summary

**Fig. 7:**
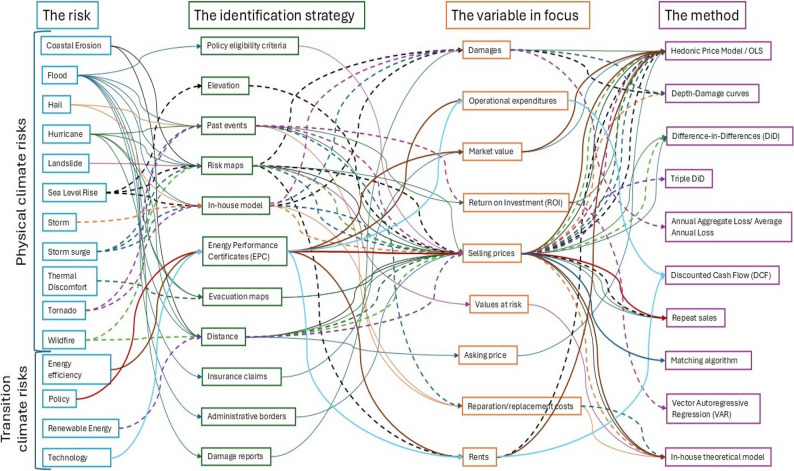
Summary of the methods, the identification strategies and the variables in focus for the physical and transition risks retrieved from the peer-reviewed literature. These elements are connected to provide a synthetic overview of the results from the map. Each study is represented by a arrow. The high number of arrows leaving from the “Flood” box indicates that many studies address this natural hazard. Similarly, the high number of arrows linking to the “Hedonic Price Model/OLS” box indicates that this method is frequently used in the literature. As an example, following the brown arrow, shows that only one study addresses policy risks, in particular “Energy Performance Certificates (EPC)”. This study examines selling prices, and employs a repeat-sales method

Figure 7 provides a visual overview of the risk, identification strategy, variable in focus and method used across the retained academic corpus.

## Conclusion

This systematic map set out to (1) identify and catalogue peer-reviewed and grey-literature evidence on how climate risks affect real-estate values, (2) describe where this evidence is concentrated across hazards, geographies, asset classes and methods, and (3) highlight clusters, gaps and priorities for future research and practice. Across both peer-reviewed and grey literature, we identified 130 relevant studies. Overall, the mapped evidence base is dominated by physical-risk studies that typically combine hazard or exposure maps with econometric capitalization approaches, while transition-risk studies remain fewer and more narrowly framed. The results of the map also provide access to evidence that can help bridge the gap between engineering-based applications, such as depth-damage curves, and observed market response. The evidence from our final corpus provides empirical estimates of the impact of climate risks on real estate markets, which can complement model-based impact functions. The parametrization and fine-tuning of models seeking to estimate these impacts can, therefore, benefit from the availability of such empirical results.

The map highlights four recurring evidence gaps. First, transition risk is covered mainly through energy-performance labels/standards and proximity effects from renewable-energy deployment, with limited attention to other transition channels such as disclosure regimes, financing and insurance repricing, shifting tenant demand, or stranded-asset dynamics. Second, explicit multi-hazard and compound/cascading risk analyses remain rare, despite their importance for decision-relevant risk assessment in real-estate markets. Third, the evidence base is geographically concentrated in the USA, with a thinner and more fragmented literature for Europe. Fourth, research is strongly skewed toward single-family housing, with comparatively limited evidence on multi-family housing and commercial real estate, where ownership structures, tenancy, financing and risk sharing differ substantially.

These patterns have practical implications. For policy and financial risk management, the evidence reinforces the importance of credible, granular exposure information: systematic production and updating of high-resolution hazard maps, especially for pluvial flooding, would improve valuation, underwriting and public investment decisions. The findings also suggest that transition policies can have distributional consequences, underscoring the need for equity-sensitive design and complementary support. Finally, because capitalization into prices depends on disclosure, salience and information frictions, strengthening property-level risk disclosure and standardising climate-adjusted valuation practices can support more consistent market pricing and better management of climate-related exposures by lenders, insurers, investors and supervisors.

## Supplementary Information

Supplementary Material 1.

Supplementary Material 2.

Supplementary Material 3.

Supplementary Material 4.

## Data Availability

The files containing all information coded from each individual study (“Coded Information.xlsx”) and the reason for full-text exclusions (“Full text exclusion.xlsx”) are available as Supplementary Files.
