# Supplementary figures and images for: Existing evidence on the impact of climate risk on real estate valuations: a systematic map
Source: Environ Evid. 2026 Jun 23;15:8. doi: 10.1186/s13750-026-00389-6 (PMC13292335; doi:10.1186/s13750-026-00389-6)

ROSES Flow Diagram for Systematic Maps. Version 1.0

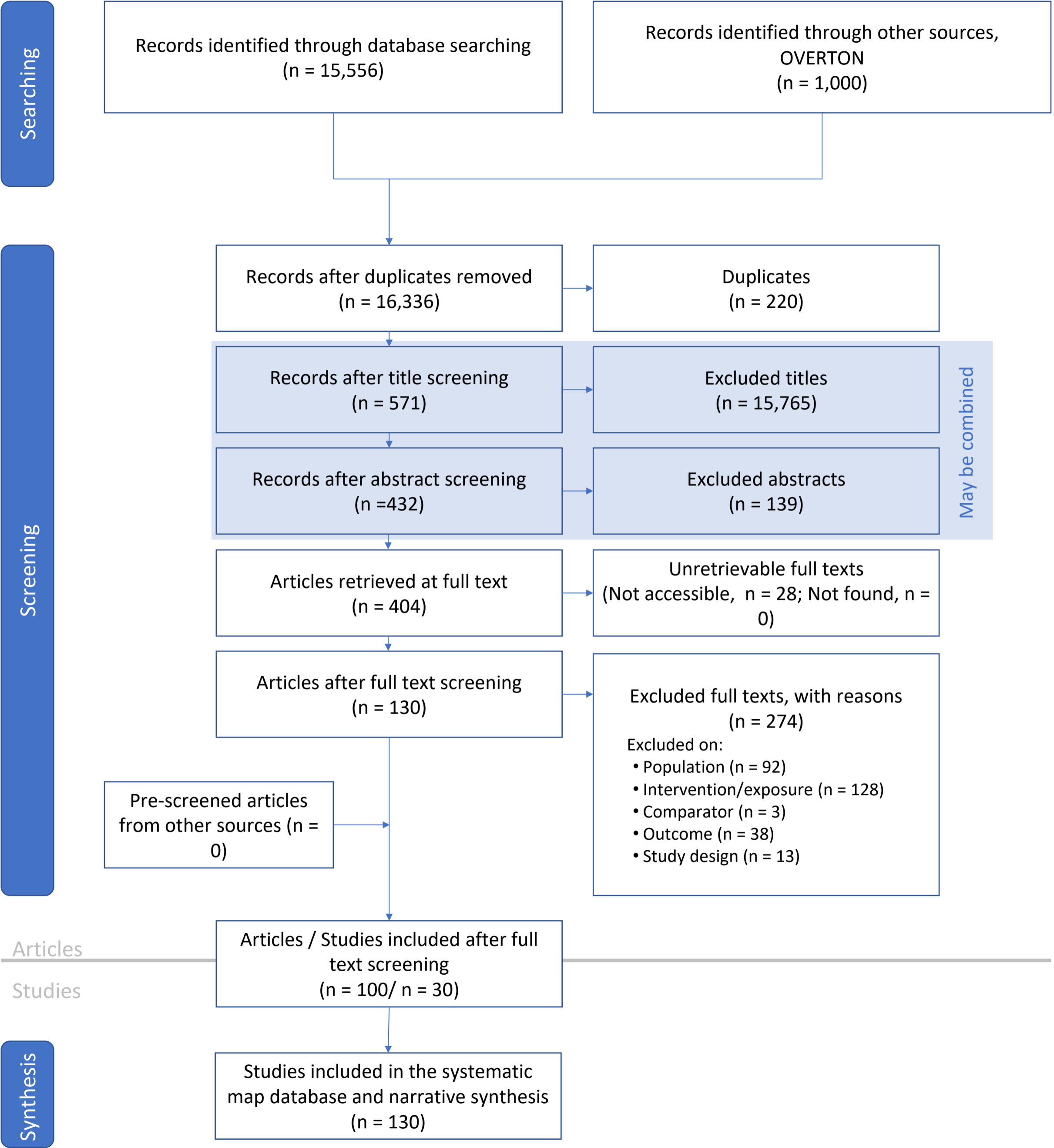

Supplement: Supplementary file 1 — Supplementary Material 1. [file 13750_2026_389_MOESM1_ESM.pdf]
