# Supplementary material for: Existing evidence on the impact of climate risk on real estate valuations: a systematic map
Source: Environ Evid. 2026 Jun 23;15:8. doi: 10.1186/s13750-026-00389-6 (PMC13292335; doi:10.1186/s13750-026-00389-6)
Supplement: Supplementary file 2 — Supplementary Material 2. [file 13750_2026_389_MOESM2_ESM.pdf]

Item number	Section/sub-section	Topic	Description	Further explanation	Checklist/meta-data	Author response	Comments
1	Title	Title	update/amendment: e.g. "...A systematic map update."	The title should normally be the same or very similar to the review question.	Meta-data	on real estate valuations: a systematic map	
2	Type of review	Type of review	systematic map amendment	See CEE Guidance on systematic mapping [1], and on amendments and updates [2]	Meta-data	systematic map	
3	Authors' contacts	Authors' contacts	provided.		Checklist	Yes	
4	Abstract	Structured summary	separate sections: Background, the context and purpose of the review, including the		Checklist	Yes	
5	Background	Background	must indicate why this study was necessary and what it aims to contribute to the field.	or exposure to the outcome.	Checklist	Yes	
6	Stakeholder engagement	Stakeholder engagement	of the question) must be described and explained (using a broad definition of		Checklist	Yes	
7	Objective of the review	Objective	Describe the primary question and secondary questions (when applicable).	linked to sources of heterogeneity (effect modifiers).	Checklist	Yes	
8		components	intervention(s)/exposure(s), comparator(s), and outcome(s).	For other question types see [4,5]	Meta-data	according to the PECO framework	
9	Methods	Protocol	Provide citation, DOI or open-access link to published protocol.	The protocol should be peer-reviewed and publicly available online (open access).	Meta-data	https://doi.org/10.1186/s13750-023-00317-y	
10		Deviations from protocol	in the protocol along with a justification.		Checklist	Yes	
11	Searches	Search strategy	searching, institutional subscriptions (or date ranges subscribed for each database),		Checklist	Yes	
12		Search string	Provide Boolean-style full search string and state the platform for which the string is formatted (e.g. Web of Science format)		Meta-data	ALL (climat* AND risk* AND (value OR economic OR financ*) AND (real AND estate OR building*)) AND (LIMIT-TO (DOCTYPE, "ar")) AND (LIMIT-TO (PUBYEAR, 2014) OR LIMIT-TO (PUBYEAR, 2015) OR LIMIT-TO (PUBYEAR, 2016) OR LIMIT-TO (PUBYEAR, 2017) OR LIMIT-TO (PUBYEAR, 2018) OR LIMIT-TO (PUBYEAR, 2019) OR LIMIT-TO (PUBYEAR, 2020) OR LIMIT-TO (PUBYEAR, 2021) OR LIMIT-TO (PUBYEAR, 2022) OR LIMIT-TO (PUBYEAR, 2023))	This is the search string we performed on Scopus. The one for Web of Science is available in the Appendix of the Protocol
13		databases	List languages used in bibliographic database searches		Meta-data	English	
14		Languages – grey literature	List languages used in organisational website searches and web-based search engines		Meta-data	English	
15		Bibliographic databases	Provide the number of bibliographic databases searched		Meta-data	3	
16		Web-based search engines	Provide the number of web-based search engines searched		Meta-data	0	
17		Organisational websites	Provide the number of organisational websites searched		Meta-data	0	
18		the search	assessed (i.e. list of benchmark articles)		Checklist	Yes	
19		Search update	Describe any update to searches undertaken during the conduct of the review	performed more than two years prior to review completion.	Checklist	Yes	
20	inclusion criteria	Screening strategy	consistency of screening decisions (at title, abstract, and full texts levels) checking must		Checklist	Yes	
21		Inclusion criteria	These must be broken down into the question key elements (e.g. relevant subject(s),		Checklist	Yes	
22	Critical appraisal	Critical appraisal strategy	assessment of individual studies and the evidence base as a whole). Describe how	Optional	Checklist	Yes	
23		Critical appraisal used in synthesis	Describe how the information from critical appraisal was used in synthesis.	Compulsory if critical appraisal performed	Checklist	n/a	
24	strategy	strategy	variables that will be extracted as meta-data and those that will be coded. Describe		Checklist	Yes	
25		Approaches to missing data	data from authors.		Checklist	Yes	
26	Data synthesis and presentation	Narrative synthesis strategy	descriptive statistics, tables (including SM database) and figures.		Checklist	Yes	
27		identification strategy	(unrepresented or underrepresented subtopics that warrant further primary research)		Checklist	Yes	
28		independence	considered within the review) in decisions regarding inclusion or critical appraisal of	prevented from unduly influencing inclusion decisions, for example by delegating tasks	Checklist	Yes	
29	Results (review findings)	Description of review process	sources and retained through each stage of the review. Must also display the number		Checklist	Yes	
30		Number of search results	conducted) prior to duplicate removal.	this will help assessment of the efficiency of the primary search string.	Meta-data	15556	
31		duplicate removal	following duplicate removal.	this will help assessment of the efficiency of the primary search string.	Meta-data	15336	
32		Full text screening excludes	Additional file containing list of and reasons for full text exclusions.		Checklist	Yes	
33		Title screening results	Provide the number of articles retained following title screening.	Optional if screening titles and abstracts together	Meta-data	571	
34		Abstract screening results	Provide the number of articles retained following abstract screening.	Optional if screening titles and abstracts together	Meta-data	432	
35		results	Provide the number of articles retained following title and abstract screening.	Optional if screening titles and abstracts separately	Meta-data		
36		Retrieval results	Provide the number of articles retrieved at full text.		Meta-data	100	peer-reviewed literature. The grey
37		Unobtainable articles	Additional file containing list of unobtainable articles.		Checklist	No	
38		Full text screening results	Provide the number of articles retained following full text screening.		Meta-data	130	Peer reviewed (100) + grey literature (30)
39		Consistency checking: screening	Results of consistency checking at all stages (screening, meta-data extraction and coding, critical appraisal) must be provided. Provide the number of titles, abstracts and full texts screened and checked for consistency by two or more reviewers as a fraction of the total (e.g. Title: 2000/20000; Abstract: 500/5000: Full text: 10/100).		Checklist	Yes	This was already done and presented in the Protocol. We here report the results from computing the kappa scores to check the consistency of the screening standards across multiple reviewers
40		Narrative synthesis	counting (tallying of studies based on results; direction or significance). Each must be		Checklist	Yes	
41		Systematic map database	Additional file containing meta-data and coding for included studies.		Checklist	Yes	
42		Limitations of the review	Discuss possible limitations in the methods used.		Checklist	Yes	
43		Limitations of the evidence base	Discuss possible limitations in the evidence base.		Checklist	Yes	
44	Conclusions	Knowledge gaps and clusters	further primary research) and knowledge clusters (well-represented subtopics that are		Checklist	Yes	
45		policy/management	evidence may inform policy/practice decision making in relation to the review/map	advocacy.	Checklist	Yes	
46		Implications for research	for increasing the reliability of study design that could improve future research.	provided it is clearly justified by the review outcome/critical appraisal of study validity.	Checklist	Yes	
47	Declarations	Competing interests	may have.		Checklist	Yes	

References

[1] James, K.L., Randall, N.P. and Haddaway, N.R., 2016. A methodology for systematic mapping in environmental sciences. Environmental Evidence, 5(1), p.7.
[2] Bayliss, H.R., Haddaway, N.R., Eales, J., Frampton, G.K. and James, K.L., 2016. Updating and amending systematic reviews and systematic maps in environmental management. Environmental Evidence, 5(1), p.20.
[3] Haddaway, N.R., Kohl, C., da Silva, N.R., Schiemann, J., Spök, A., Stewart, R., Sweet, J.B. and Wilhelm, R., 2017. A framework for stakeholder engagement during systematic reviews and maps in environmental management. *Environmental Evidence* , *6* (1), p.11.
[4] Collaboration for Environmental Evidence. 2018. Guidelines and Standards for Evidence synthesis in Environmental Management. Version 5.0. www.environmentalevidence.org/information-for-authors.
[5] Leeds Institute of Health Sciences. https://medhealth.leeds.ac.uk/info/639/information_specialists/1500/search_concept_tools. Accessed 12/11/2017.
